# An Investigation on Phenolic and Antioxidant Capacity of Under-utilized Food Legumes Consumed in China

**DOI:** 10.3390/foods9040438

**Published:** 2020-04-06

**Authors:** Yaqian Zhang, Maninder Meenu, Hansong Yu, Baojun Xu

**Affiliations:** 1 College of Food Science and Engineering, Jilin Agricultural University, Changchun 130118, China; yaqianzhang9@gmail.com; 2 Food Science and Technology Program, Beijing Normal University-Hong Kong Baptist University United International College, Zhuhai 519087, China; meenu_maninder@yahoo.com (M.M.); baojunxu@uic.edu.hk (B.X.)

**Keywords:** under-utilized food legumes, phenolics, flavonoids, tannins, antioxidant ability, high-performance liquid chromatography (HPLC)

## Abstract

China is a major producer of various kinds of food legumes. Some of the under-utilized food legumes are consumed by the local society from different areas in China. The antioxidant capacity and phytochemical profile of these under-utilized food legumes haven’t been investigated until now. In this study, colorimetric and high-performance liquid chromatography was employed to explore the antioxidant capacity and phytochemical profile of 21 under-utilized food legumes. Different legumes under investigation exhibit a wide range of variations in their total phenolic content (TPC), total flavonoids content (TFC) and condensed tannins content (CTC). Among all the legume samples, the velvet bean from Hechi, Guangxi exhibited the highest antioxidant capacity while the white flat bean from Shangrao, Shanxi presented the least antioxidant capacity. Gallic acid was observed to be a major phenolic acid and its content in the velvet bean was significantly higher compared to the other legume samples explored in this study. The composition of flavonoids was different among all the legumes. Kaempferol was observed to be the most predominant flavonoid. The findings of this study will be beneficial for plant breeders, food scientists and consumers for the better selection of germplasm with a high level of phytochemicals that in turn possess maximum health benefits.

## 1. Introduction

Food legumes play an important role in the traditional diets of many regions throughout the world. They are a rich source of protein, dietary fiber and are low in fat. These food legumes also contain multifarious micronutrients and phytochemicals which are reported to present various health-promoting benefits [1]. Food legumes are usually consumed together with other food materials in the form of salads, sweet foods, and so on. The legumes are reported to exhibit protective and therapeutic effects [2] as well as health benefits associated with the prevention of chronic diseases [3] such as breast cancer, cardiovascular diseases and cataract development. These health benefits are attributed to the high content of antioxidants present in the food legumes [4].

Free radicals are involved in the etiology of various diseases by damaging various biochemicals such as DNA, proteins, carbohydrates, unsaturated lipids, and micronutrients [3]. It is widely known that the antioxidants from plants and plant-based foods are responsible for the reduction of radical-related pathogenicity [5]. Legumes are also reported to exhibit strong antioxidant capacities due to the presence of a wide range of various types of phenolic compounds [3,4]. Phenolic compounds are the small molecules characterized by having at least one phenol group in their structure. These compounds can mainly be divided into phenolic acids and flavonoids [6]. In addition, bioactive peptides from legume grains are also reported to exhibit nutraceutical and therapeutic applications [7].

There are various types of food legumes cultivated in China. China has a vast territory with complex ecological conditions suitable for the production of various crops throughout the year. Food legumes are also cultivated throughout the country. Among all the food legumes, soybean, broad bean, peas, mung bean, common bean, and adzuki bean are the major legume species. Whereas the area under production for the lentils, chickpeas, and some other legumes is comparatively less cultivated [8]. Furthermore, some of the local edible legumes have been cultivated in different provinces, such as velvet bean in Guangxi province, stone bean in Sichuan province and large zebra bean in Guangdong province. These food legumes are considered to be under-utilized food legumes in China as these food legumes are only known and consumed by the local people of a particular province. These special beans contain a high level of nutrients and some of them can also be used in traditional Chinese medicines [9]. 

These legume species were neglected by researchers, marketing systems and conservation [10]. These varieties have been gradually replaced by the high yielding stable crops produced by employing modern breeding techniques. In the current scenario, these legumes are not able to meet modern quality standards such as uniformity and others due to the lack of technological interventions [11]. Thus, these legumes are less preferred by consumers and less competitive in market. However, lack of technological intervention results in the production of secondary metabolite phytochemicals in these legume crops that helps these crops to survive under adverse environmental and extreme stress conditions [10,11]. 

Previously, some of the researchers have studied the methods of cultivation and macronutrients present in these special beans. However, only one study has explored the phenolic content and antioxidant capability of eight kinds of special beans [12]. The main focus of that study was to explore the total flavonoid content of these beans, whereas the phenolic profile of these legumes was not investigated.

Thus, in the present study, 21 types of under-utilized food legumes were collected from different geographical areas of China and their total phenolic content (TPC), total flavonoids content (TFC), condensed tannins content (CTC), and antioxidant capacities were determined by colorimetric methods. In addition, high-performance liquid chromatography (HPLC) was also employed to determine the phenolic acids and flavonoids of these legume samples. In addition to the 21 types of food legumes, the TPC, TFC, CTC, antioxidant capacity and phenolic profile of two types of adzuki bean were also explored for the comparison purpose. The output of this study will provide a baseline data related to phytochemical profile and antioxidant activity of these under-utilized legumes collected from different geographical areas of China that will be useful for the consumer, plant breeders and food scientists for the development and cultivation of phytochemical rich legume varieties, extraction of pharmacologically important components and development of novel food products.

## 2. Materials and Methods 

### 2.1. Legume Samples and Moisture Content Analysis

A total of 21 types of legumes (as shown in Supplementary Table S1) were collected from different provinces in China. All of the samples were dry food legumes and stored at room temperature for two weeks before chemical analysis. The moisture content of legumes samples was analyzed by using a moisture analyzer (MA-150, Sartorius, Genthin, Germany). 

### 2.2. Chemicals and Reagents 

(+)-Catechin, 2,2’-azino-bis (3-ethylbenzothiazoline-6-sulfonic acid) (ABTS), 2,4,6-tri (2-pyridyl) -s-triazine (TPTZ), 6-hydroxy-2,5,7,8-tetramethlchroman-2 -carboxylic acid (Trolox), gallic acid, Folin-Ciocalteu reagent, aluminum chloride hexahydrate (AlCl_3_·6H_2_O), methanol, vanillin, concentrated hydrochloric acid, potassium persulphate (K_2_S_2_O_8_), phosphate buffer saline (PBS), sodium acetate trihydrate, glacial acetic acid, ferric chloride (FeCl_3_·6H_2_O), ferrous sulfate (FeSO_4_·7H_2_O) and acetone were purchased from Tianjin Damao Chemical Reagent Co., Ltd. (Tianjin, China). Absolute ethanol was procured from Tianjin Fuyu Fine Chemical Co., Ltd. (Tianjin, China). Trifluroacetic acid (TFA), acetonitrile (HPLC grade), and methanol (HPLC grade) were purchased from Sigma-Aldrich Co., Ltd. (Shanghai, China). 

### 2.3. Extraction of Legume Samples

The legume sample was ground using an electric grinding machine and passed through a 60-mesh sieve. The extraction of the legume samples was carried out according to a previously described method by Xu and Chang [13]. Briefly, 0.5 g legume powder was extracted using 5 mL extraction solution (acetone:water:acetic acid = 70:29.5:0.5) at room temperature for 3 hours by shaking at 300 rpm using an orbital shaker. The solution was separated by centrifugation for 10 min at 3000 rpm. The residue was extracted again using the same procedure and both the extracts were mixed. Extraction of each legume sample was carried out in triplicate.

### 2.4. Colorimetric Analysis of 23 Legumes

Colorimetric methods described by Xu and Chang [13] were employed to assess total phenolics content (TPC), total flavonoids content (TFC), condensed tannins content (CTC), ferric reducing antioxidant capacity (FRAP), and ABTS free radical scavenging capacity (ABTS) of various legume samples. The TPC was expressed as gallic acid equivalents (mg of GAE/g sample) on a dry weight basis. The TFC was expressed as catechin equivalents (µg of CAE/g sample) on a dry weight basis. The CTC was expressed as catechin equivalents (µg of CAE/g sample) on a dry weight basis. The FRAP value was expressed as mmol of Fe^2+^ equivalents per 100 g of sample (mmol Fe^2+^ E/100 g sample) on a dry weight basis. The ABTS was expressed as Trolox equivalents (µmol TE/g sample) on a dry weight basis.

### 2.5. Determination of Color Values

The color values of 23 legume samples were recorded by using a Minolta Color Difference Meter (Model CR-400, Konica Minolta, Osaka, Japan) using Hunter scale for *L*, *a* and *b*. Standard white plate (Y = 93.5, x = 0.3114, y = 0.3190) was used to calibrate the instrument. Results were expressed as tri-stimulus values *L*: lightness (0 = black, 100 = white), *a* (–*a* = greenness, +*a* = redness), and *b* (–*b* = blueness, +*b* = yellowness). 

### 2.6. HPLC Determination of 23 Legumes

The extraction of legume samples was carried out according to the method described by Xu and Chang [14] with a slight modification. Briefly, 10 mL of extraction solution (methanol:water:acetic acid:BHT = 85:15:0.5:0.2) was added into a tube with 1 g sample powder. Extraction was performed for 4 hours by shaking the reaction mixture at 250 rpm. Then, the solution was separated by centrifugation at 10,000 rpm for 15 min. The extraction was repeated again and the two-time solutions were combined together. Finally, the extract was subjected to vacuum drying at 40 °C and re-dissolved in 1.5 mL of 25% methanol followed by filtration through Whatman #1 filter paper. For every legume sample, the extraction was carried out in triplicate.

### 2.7. Determination of Free Phenolic Acids

The quantification of free phenolic acids in legume samples was carried out by following the method described by Xu and Chang [14]. The analysis of free phenolic acids was conducted by employing Waters HPLC (e2695 Separations Modulek, Milford, MA, USA) equipped with ZORBAX SB-C18 column (4.6 mm × 250 mm, 5-Micron) at 40 °C and Waters 2998 photodiode array detector. TFA (0.1%) and methanol were used as mobile phase A and mobile phase B, respectively for elution with a flow rate of 0.7 mL/min. The gradient of the solvent system was 95% of mobile phase A at 0 min, 70% at 50 min and 65 min, 0% at 66 min and 76 min, and 95% at 77 min.

### 2.8. Determination of Flavonoids

The method reported by Xu and Chang [14] was employed for the determination of flavonoids in legumes samples. A Waters HPLC system (e2695 Separations Modulek, Milford, MA, USA) equipped with YMC-Pack ODS-AM column (250 mm × 4.6 mm) at 4 °C was used for the separation of various flavonoids present in samples. TFA (0.1%) and acetonitrile were used as mobile phase A and mobile phase B, respectively, for elution. The flow rate was set at 1 mL/min. The gradient of the solvent system followed by 90% of mobile phase A at 0 min, 70% at 20 min, 60% at 30 min, 50% at 50 and 55 min, 80% at 59 min, and 90% at 60 min.

### 2.9. Statistical Analysis 

All assays were performed in triplicate. The results were reported as mean ± standard deviations. The significant differences between the mean values of various parameters were analyzed through Duncan’s multiple range test. The Pearson correlation analysis was used to analyze the correlation among variables. Probability values of less than 0.05 were considered to be significant. All statistical analyses were performed by employing SPSS Statistic (version 24).

## 3. Results

### 3.1. Total Phenolic Content (TPC) of Under-Utilized Food Legumes

The TPC values (expressed as mg GAE/g) of 23 types of legume samples are shown in Table 1. The TPC values of all of the samples ranged from 0.74 mg GAE/g to 56.63 mg GAE/g. Among all the samples, the velvet bean from Hechi, Guangxi (56.63 mg GAE/g) exhibited significantly higher phenolic content. In addition to the velvet bean, the spotted cowpea from Chuzhou, Anhui (14.62 mg GAE/g), small adzuki bean from Jixi, Heilongjiang (13.29 mg GAE/g), stone bean from Dazhou, Sichuan (13.27 mg GAE/g), small black bean from Liangshan, Sichuan (12.79 mg GAE/g), mosaic bean from Jinzhong, Shanxi (11.16 mg GAE/g) and green adzuki bean from Diqing Tibetan Autonomous Prefecture, Yunnan (10.84 mg GAE/g) presented relatively higher total phenolic content, while the white flat bean from Shangrao, Shanxi (0.71 mg GAE/g), white kidney bean from Diqing Tibetan Autonomous Prefecture, Yunnan (0.74 mg GAE/g) and small white bean from Chengdu, Sichuan (0.74 mg GAE/g) exhibited very low phenolic content, and 13 other types of legumes had phenolic content ranging between 1.04 mg GAE/g and 9.62 mg GAE/g.

The colorimetric method used in this study revealed that most of the legumes exhibited a high level of the total phenolics, in which the highest TPC value of velvet bean from Hechi, Guangxi (56.63 mg GAE/g) was 79-folds higher than the white flat bean from Shangrao, Shanxi (0.71 mg GAE/g) which presented the lowest TPC value. In general, significant differences (*p* < 0.05) were observed among the TPC values of most of the food legumes under investigation. However, no significant difference was found in among the TPC value of the small adzuki bean (Jixi, Heilongjiang), stone bean (Dazhou, Sichuan) and small black bean (Liangshan, Sichuan), between the green adzuki (Diqing Tibetan Autonomous Prefecture, Yunnan) and mosaic bean (Jinzhong, Shanxi), between the small zebra bean (Heze, Shandong) and white kidney bean (Diqing Tibetan Autonomous Prefecture, Yunnan), between the light speckled bean (Kunming, Yunnan) and black eyed peas (Zhaoqing, Guangdong) and among the white flat bean (Shangrao, Shanxi), large white kidney (Diqing Tibetan Autonomous Prefecture, Yunnan), white kidney (Diqing Tibetan Autonomous Prefecture, Yunnan), and small white bean (Chengdu, Sichuan).

### 3.2. Total Flavonoid Content (TFC) of Under-Utilized Food Legumes

The TFC values (expressed as mg CAE/g) of 23 types of legumes are presented in Table 1. Among the legume samples, the velvet bean from Hechi, Guangxi (34.50 mg CAE/g) exhibited the highest flavonoid content, which was 3-fold higher than the mosaic bean from Jinzhong, Shanxi (10.39 mg CAE/g) that presented the second highest TFC value among all the legume samples under investigation. As shown in Table 1, the white flat bean from Shangrao, Shanxi (1.05 mg CAE/g), large white kidney from Diqing Tibetan Autonomous Prefecture, Yunnan (1.34 mg CAE/g), white kidney bean from Diqing Tibetan Autonomous Prefecture, Yunnan (1.46 mg CAE/g) and small white bean from Chengdu, Sichuan (1.27 mg CAE/g) exhibited significantly lower levels of TFC, while 17 other types of legumes exhibited TFC ranging from 2.25 mg CAE/g to 7.91 mg CAE/g. 

The TFC values of the velvet bean (34.50 mg CAE/g) were observed to be 32-fold higher compared to the legume sample white flat beans that presented the lowest TFC value (1.05 mg CAE/g). On the whole, most of the legume samples presented significant differences in their TFC value (*p* < 0.05). However, no significant differences were observed in the case of the small zebra bean (Heze, Shandong) and mosaic bean (Jinzhong, Shanxi), between the milky flower bean (Luohe, Henan) and light speckled bean (Kunming, Yunnan), and also no significant differences were found between the large white kidney bean (Diqing Tibetan Autonomous Prefecture, Yunnan) and white kidney bean (Diqing Tibetan Autonomous Prefecture, Yunnan).

### 3.3. Condensed Tannin Content (CTC) of Under-Utilized Food Legumes

The CTC values (expressed as mg CAE/g) of 23 types of legume samples are shown in Table 1. The CTC values of all of the legume samples ranged from 0.98 mg CAE/g in the case of the white flat bean (Shangrao, Shanxi) to 12.95 mg CAE/g in case of the stone bean (Dazhou, Sichuan, China). The stone bean (12.95 mg CAE/g) and spotted cowpea from Chuzhou, Anhui (12.01 mg CAE/g) exhibited a relatively higher value of CTC, whereas the white flat bean from Shangrao, Shanxi presented the lowest CTC value. The other 20 types of legume samples presented CTC values in the range of 1.52 mg CAE/g to 9.47 mg CAE/g.

Overall, the legume samples under investigation presented significant differences (*p* < 0.05) in CTC values. However, no significant differences were found between the red kidney bean (Kunming, Yunnan, China) and mosaic bean (Jinzhong, Shanxi, China), among the pinto bean (Kunming, Yunnan, China), pinto kidney bean (Nujiang Lisu Autonomous Prefecture, Yunnan, China) and small black bean (Liangshan, Sichuan, China), between the adzuki bean (Mudanjiang, Heilongjiang, China) and pinto bean (Kunming, Yunnan), between the large zebra bean (Shaoguan, Guangdong) and small zebra bean (Heze, Shandong, China), between the light speckled bean (Kunming, Yunnan, China) and velvet bean (Hechi, Guangxi), between the white kidney bean (Diqing Tibetan Autonomous Prefecture, Yunnan, China) and black eyed pea (Zhaoqing, Guangdong, China), and between the large white kidney bean (Diqing Tibetan Autonomous Prefecture, Yunnan, China) and small white bean (Chengdu, Sichuan, China).

### 3.4. Ferric Reducing Antioxidant Capacity (FRAP) of Under-Utilized Food Legumes

The values of FRAP (expressed as mmol Fe^2+^E/100 g) of 23 types of legumes are presented in Table 1. Among the legume samples, the velvet bean from Hechi, Guangxi (102.82 mmol Fe^2+^ E/100 g) exhibited the highest FRAP value, which was around 4-fold higher than the spotted cowpea from Chuzhou, Anhui (23.24 mmol of Fe^2+^E/100g) and stone bean from Dazhou, Sichuan (23.23 mmol of Fe^2+^E/100g). The small adzuki bean (Jixi, Heilongjiang, China), broad bean (Kunming, Yunnan, China), green adzuki bean (Diqing Tibetan Autonomous Prefecture, Yunnan), mosaic bean (Jinzhong, Shanxi, China), small zebra bean (Heze, Shandong), small black bean (Liangshan, Sichuan), red kidney bean (Kunming, Yunnan, China), adzuki bean (Mudanjiang, Heilongjiang, China), pinto bean (Kunming, Yunnan, China), pinto kidney bean (Nujiang Lisu Autonomous Prefecture, Yunnan) and red pinto bean (Anqing, Anhui, China) exhibited relatively higher FRAP values of 19.27, 18.83, 17.38, 16.94, 15.48, 15.33, 12.92, 11.81, 11.59, 11.45, and 10.38 mmol of Fe^2+^E/100g, respectively, while the white flat bean from Shangrao, Shanxi (0.59 mmol of Fe^2+^E/100g), large white kidney bean from Diqing Tibetan Autonomous Prefecture, Yunnan (0.90 mmol of Fe^2+^E/100g), white kidney bean from Diqing Tibetan Autonomous Prefecture, Yunnan (0.78 mmol of Fe^2+^E/100g) and small white bean from Chengdu, Sichuan (0.96 mmol of Fe^2+^E/100g) presented comparatively very low FRAP values. The FRAP value of another five legumes samples ranged from 2.99 mmol of Fe^2+^E/100g to 9.66 mmol of Fe^2+^E/100g.

On the whole, most of the legume samples presented significant differences (*p* < 0.05) in their FRAP values. No significant differences were found between the FRAP values of the stone bean (Dazhou, Sichuan) and spotted cowpea (Chuzhou, Anhui, China), among the small adzuki bean (Jixi, Heilongjiang) and broad bean (Kunming, Yunnan), between the green adzuki bean (Diqing Tibetan Autonomous Prefecture, Yunnan, China) and mosaic bean (Jinzhong, Shanxi, China), between the small zebra bean (Heze, Shandong) and small black bean (Liangshan, Sichuan, China), between the large zebra bean (Shaoguan, Guangdong, China) and small zebra bean (Heze, Shandong, China), and between the light speckled bean (Kunming, Yunnan) and black eyed pea (Zhaoqing, Guangdong, China). No significant difference was observed among the white flat bean (Shangrao, Shanxi), large white kidney bean (Diqing Tibetan Autonomous Prefecture, Yunnan, China), white kidney bean (Diqing Tibetan Autonomous Prefecture, Yunnan, China) and small white bean (Chengdu, Sichuan, China).

### 3.5. ABTS Free Radical Scavenging Activity (ABTS) of Under-Utilized Food Legumes

The values of ABTS (expressed as μmol TE/g) of 23 types of legumes are presented in Table 1. The highest ABTS value was recorded in case of the velvet bean from Hechi, Guangxi (298.7 μmol TE/g) which was two times higher than the broad bean from Kunming, Yunnan (140.6 μmol TE/g) that presented the second highest ABTS value among all the legume samples under investigation. As shown in Table 1, the spotted cowpea from Chuzhou, Anhui (124.9 μmol TE/g), stone bean from Dazhou, Sichuan (122.7 μmol TE/g) and small adzuki bean from Jixi, Heilongjiang (104.2 μmol TE/g) also presented significantly higher values for ABTS, whereas the white flat bean from Shangrao, Shanxi (5.41 μmol TE/g), large white kidney bean from Diqing Tibetan Autonomous Prefecture, Yunnan (9.04 μmol TE/g), white kidney bean from Diqing Tibetan Autonomous Prefecture, Yunnan (7.74 μmol TE/g) and small white bean from Chengdu, Sichuan (8.76 μmol TE/g) presented relatively low radical scavenging activity as accessed by ABTS method. The ABTS values for 14 other types of legume samples varied from 17.4 μmol TE/g to 96.9 μmol TE/g.

Overall, all of the legume samples under investigation presented significant differences (*p* < 0.05) in ABTS values. However, no significant differences were found between the stone bean (Dazhou, Sichuan) and spotted cowpea (Chuzhou, Anhui), among the adzuki bean (Mudanjiang, Heilongjiang) and pinto bean (Kunming, Yunnan), between the light speckled bean (Kunming, Yunnan) and black-eyed pea (Zhaoqing, Guangdong), and also no significant difference was observed among the white flat bean (Shangrao, Shanxi), large white kidney bean (Diqing Tibetan Autonomous Prefecture, Yunnan), white kidney bean (Diqing Tibetan Autonomous Prefecture, Yunnan) and small white bean (Chengdu, Sichuan).

### 3.6. Color Value

The *L*, *a*, *b* values of legume samples are shown in Table 2. The *L* value of 23 legumes ranged from 79.7 in the case of the small black bean (Liangshan, Sichuan) to 99.2 in the case of the large white kidney bean (Diqing Tibetan Autonomous Prefecture, Yunnan). The *a* values of legumes varied from -4.13 in the case of the small black bean (Liangshan, Sichuan) to 3.74 in the pinto bean (Kunming, Yunnan). The *b* value of legumes was observed to be in a range from 0.9 in the case of the stone bean (Dazhou, Sichuan) to 15.75 in the case of the small black bean (Liangshan, Sichuan).

### 3.7. Correlations between Phytochemicals, Antioxidant Capacities and Color Value of 23 Legumes

The correlation among phytochemicals, antioxidant capacities and color value of 23 legumes are presented in Table 3. A significantly higher positive correlation was observed between TPC and TFC at the level of 0.01 (*r* = 0.99), while the correlation between TPC and CTC (*r* = 0.161), and TFC and CTC (*r* = 0.104) was not obvious. Among the antioxidant abilities of all legumes, FRAP value exhibited a significantly higher correlation with ABTS at the level of 0.01 (*r* = 0.94). In addition, a significant positive correlation between TPC and FRAP (*r* = 0.995), TPC and ABTS (*r* = 0.944), TFC and FRAP (*r* = 0.987), and TFC and ABTS (*r* = 0.912) was also observed. A poor correlation was observed between CTC and FRAP values (*r* = 0.118), and between CTC and ABTS values (r = 0.412).

### 3.8. Free Phenolic Acids of Under-Utilized Food Legumes

The free phenolic acid content of all 23 types of legume samples is presented in Table 4. Gallic acid, *p*-hydroxybenzoic acid, vanillic acid, *p*-coumaric acid, gentistic acid, ferulic acid, 2,3,4-trihydroxybenzoic acid, syringic acid, protocatechualdehyde and vanillin were detected in the majority of legume samples under investigation, while chlorogenic acid, sinapic acid and salicylic acid substances were only observed in a few samples.

As shown in Table 4, among 14 phenolic acids, gallic acid was the most predominant phenolic acid, which was discovered in 22 samples out of 23 legume samples. Among the 22 samples, velvet bean from Hechi, Guangxi (479.26 μg/g) exhibited the highest gallic acid content, which was 13-fold higher than the small black bean from Liangshan, Sichuan (28.64 μg/g) and 19-fold higher than the broad bean from Kunming, Yunnan (24.55 μg/g) that exhibited second and third highest level of gallic acid. Among other legume samples, the gallic acid varied from 0.39 μg/g to 14.52 μg/g.

The second predominant free phenolic acids were *p*-hydroxybenzoic acid, vanillic acid and *p*-coumaric acid, which were found in 15 different legume samples. Among the legume samples, the content of *p*-hydroxybenzoic acid ranged from 0.21 μg/g to 6.9 μg/g, in which the small zebra bean from Heze, Shandong exhibited the highest content while the white flat bean from Shangrao, Shanxi possessed the lowest content. For vanillic acid, the white flat bean from Shangrao, Shanxi (14.85 μg/g) and the large zebra bean from Shaoguan, Guangdong (12.36 μg/g) presented relatively high content, while the broad bean from Kunming, Yunnan (0.33 μg/g) and the black-eyed pea from Zhaoqing, Guangdong (0.14 μg/g) showed extremely low content. In case of other legume samples, vanillic acid ranged from 2.07 μg/g to 8.85 μg/g. In terms of *p*-coumaric acid, the small black bean from Liangshan, Sichuan (6.53 μg/g) presented the highest content followed by the white flat bean from Shangrao, Shanxi (5.88 μg/g), while the red kidney from Kunming, Yunnan showed the lowest *p*-coumaric acid content, and in the case of other legume samples, *p*-coumaric acid varied from 1.30 μg/g to 3.01 μg/g.

Gentistic acid and ferulic acid were the third predominant free phenolic acid and were detected in 14 legume samples. The spotted cowpea from Chuzhou, Anhui (18.94 μg/g) exhibited the highest amount of gentistic acid, while the stone bean from Dazhou, Sichuan (0.81 μg/g), red kidney bean from Kunming, Yunnan (0.64 μg/g) and broad bean from Kunming, Yunnan (0.11 μg/g) showed very low content of gentistic acid. The red kidney (17.98 μg/g) presented the highest content of ferulic acid, while the small round pinto bean from Nujiang Lisu Autonomous Prefecture, Yunnan exhibited the lowest content, and in the case of others legume samples, the ferulic acid content ranged from 1.37 μg/g to 4.23 μg/g.

2,3,4-Trihydroxybenzoic acid and syringic acid were detected in 13 legume samples. The range of 2,3,4-trihydroxybenzoic acid varied significantly from 1.58 μg/g (large zebra bean from Shaoguan, Guangdong) to 6.13 μg/g (velvet bean from Hechi, Guangxi). The syringic acid ranged from 0.2 μg/g (black-eyed pea from Zhaoqing, Guangdong) to 6.08 μg/g (large kidney bean from Diqing Tibetan Autonomous Prefecture, Yunnan).

Protocatechuic acid, protocatechualdehyde and vanillin were detected in 12 samples. Protocatechuic acid varied from 0.16 μg/g in the case of the red pinto bean (Kunming, Yunnan) to 4.68 μg/g in the case of the large zebra bean (Shaoguan, Guangdong). However, the protocatechualdehyde varied significantly from 0.11 μg/g (large zebra bean) to 29.71 μg/g (velvet bean). The range of vanillin was observed to be 1.16 μg/g in the case of the large zebra bean to 219.19 μg/g in the case of the velvet bean.

Sinapic acid was identified in 10 legume samples; among these samples, the small round pinto bean from Nujiang Lisu Autonomous Prefecture, Yunnan (1.94 μg/g) exhibited the highest sinapic acid content while the light speckled bean from Kunming, Yunnan (0.21 μg/g) showed the lowest content. In addition, the salicylic acid was discovered in only four samples, in which the small zebra bean from Heze, Shandong (12.55 μg/g) contained the highest content and adzuki bean from Mudanjiang, Heilongjiang presented the lowest content (0.59 μg/g).

The sum of free phenolic acids in each legume sample is presented in Table 4. Among all these legume samples, the velvet bean from Hechi, Guangxi exhibit the highest free phenolic acids content, followed by the broad bean from Kunming, Yunnan (194.67 μg/g), small adzuki bean from Jixi, Heilongjiang (113.07 μg/g) and adzuki bean from Mudanjiang, Heilongjiang (102.76 μg/g), while the small round pinto bean from Nujiang Lisu Autonomous Prefecture, Yunnan (14.52 μg/g) presented the lowest value for the sum of free phenolic acids.

Overall, a significant difference (*p* < 0.05) was observed in the gallic acid content of legume samples under investigation. However, no significant difference was observed in the gallic acid content of the adzuki bean (Mudanjiang, Heilongjiang), pinto bean (Kunming, Yunnan), milky flower bean (Luohe, Henan), spotted cowpea (Chuzhou, Anhui), white flat bean (Shangrao, Shanxi), pinto kidney bean (Nujiang Lisu Autonomous Prefecture, Yunnan), white kidney bean (Diqing Tibetan Autonomous Prefecture, Yunnan), stone bean (Dazhou, Sichuan), light speckled bean (Kunming, Yunnan), red kidney bean (Kunming, Yunnan), green adzuki (Diqing Tibetan Autonomous Prefecture, Yunnan) and black-eyed pea (Zhaoqing, Guangdong). In addition, no significant difference was detected between the small adzuki bean (Jixi, Heilongjiang) and mosaic bean (Jinzhong, Shanxi), large kidney bean (Diqing Tibetan Autonomous Prefecture, Yunnan) and small round pinto bean (Nujiang Lisu Autonomous Prefecture, Yunnan), red pinto bean (Anqing, Anhui) and large zebra bean (Shaoguan, Guangdong).

No significant difference (*p* > 0.05) was observed in the ferulic acid of legume samples except for the red kidney beans from Kunming, Yunnan (17.98 μg/g) which exhibited a significant difference in ferulic acid content compared to the other samples. Salicylic acid was found in only four legume samples and there was a significant difference (*p* < 0.05) among the salicylic acid content of these samples. For other individual phenolic acids, a significant difference (*p* < 0.05) was observed among some of the food legumes, while no significant difference was found in the case of some other legume samples. For example, on the whole, a significant difference was observed in the case of 2,3,4-trihydroxybenzoic acid values of various legume samples. However, no significant difference was observed (*p* > 0.05) between the pinto bean (Kunming, Yunnan) and red pinto bean (Anqing, Anhui), and among the pinto kidney bean (Nujiang Lisu Autonomous Prefecture, Yunnan) and red kidney bean (Kunming, Yunnan).

### 3.9. Flavonoids of Under-Utilized Food Legumes

The flavonoids profile of all 23 types of legume samples under investigation is presented in Table 5. Among the 12 flavonoids explored, hesperitin was the most predominant flavonoid, which was found in most of the legume samples, and tangeretin was observed to be the second most predominant flavonoid among the legume samples. Naringenin, apigenin, kaempferol, chrysin, galangin, and tangeretin were not detected and others were found to be present in a very low quantity.

As shown in Table 5, hesperitin was discovered in 19 out of 23 legume samples. Among the 19 samples, the small black bean from Liangshan, Sichuan (38.1 μg/g) had the highest content of hesperitin while the light speckled bean from Kunming, Yunnan (4.56 μg/g) had the lowest content. Tangeritin was detected in 10 out of 23 legume samples and ranged from 2.69 μg/g (small round pinto bean from Nujiang Lisu Autonomous Prefecture, Yunnan) to 5.63 μg/g (red kidney from Kunming, Yunnan).

Quercitrin and hesperidin were found in nine legume samples. The small adzuki bean (21.69 μg/g) exhibited the highest amount of quercitrin and hesperidin, whereas the milky flower (3.83 μg/g) presented the lowest content. Naringin was detected in only six samples, while the small adzuki bean from Jixi, Heilongjiang (76.79 μg/g) contained the highest content and the red pinto bean from Anqing, Anhui (13.88 μg/g) exhibited the lowest content. Myricetin and morin were only observed in three and two samples, respectively. The small black bean from Liangshan, Sichuan was observed to contain the highest quantity of myricetin (63.32 μg/g) and morin (39.27 μg/g), whereas a significant amount of quercetin and luteolin was only found in the small black bean (5.8 μg/g).

The sum of flavonoids in all of the legume samples is shown in Table 5. The small black bean from Liangshan, Sichuan (149.86 μg/g) presented the highest value for the sum of flavonoids, followed by the small adzuki bean from Jixi, Heilongjiang (149.86 μg/g) and the black-eyed pea from Zhaoqing, Guangdong (89.52 μg/g). The large kidney bean from Diqing Tibetan Autonomous Prefecture, Yunnan exhibited the lowest value for the sum of flavonoids.

In this study, seven different types of flavonoid were detected in the legume samples under investigation. For each individual flavonoid, a significant difference (*p* < 0.05) was found within most of the legume samples. However, for hesperitin, no significant difference (*p* > 0.05) was observed between the milky flower bean (Luohe, Henan) and small zebra bean (Heze, Shandong), and among the red kidney bean (Kunming, Yunnan), green adzuki bean (Diqing Tibetan Autonomous Prefecture, Yunnan) and mosaic bean (Jinzhong, Shanxi). In the case of tangeretin, no significant difference was found between the pinto bean (Kunming, Yunnan) and white flat bean (Shangrao, Shanxi), and the large kidney bean (Diqing Tibetan Autonomous Prefecture, Yunnan) and small round pinto bean (Nujiang Lisu Autonomous Prefecture, Yunnan).

## 4. Discussion

### 4.1. Phenolic Compounds in Under-Utilized Food Legumes

Previously, Xu and Chang [15] have reported the TPC of red kidney beans, small black beans, adzuki beans and black-eyed peas from the USA. In this study, the TPC value of the red kidney bean from Kunming, Yunnan (9.18 mg GAE/g) and small black bean from Liangshan, Sichuan (12.79 mg GAE/g) was slightly higher compared to that previously mentioned by Xu and Chang [15], while the TPC value for the black eyed pea from Zhaoqing, Guangdong (2.34 mg GAE/g) was similar to the value reported by Xu and Chang [15]. The TPC value of the adzuki bean from Mudanjiang, Heilongjiang measured in this study (13.29 mg GAE/g) was relatively close to the TPC value of the adzuki bean reported by Xu and Chang [15]. According to Woo et al. [16], the TPC values of different types of adzuki beans cultivated by different methods ranged from 6.21 mg GAE/g to 10.86 mg GAE/g. In this study, the TPC value of the adzuki bean was 8.9 mg GAE/g, which falls within the range as reported earlier [17].

Xu and Chang [15] have also reported the CTC values of the four types of legumes. In this study, the CTC values of the red kidney bean from Kunming, Yunnan (8.44 mg CAE/g), small black bean (7.66 mg CAE/g) and black-eyed peas from Zhaoqing, Guangdong (2.68 mg CAE/g) were slightly higher or lower compared to the finding of Xu and Chang’s study [15]. CTC values of adzuki beans were lower compared to the CTC value reported by Xu and Chang [15]. The slight discrepancy between the results is acceptable as the sources of the legume samples explored in these two studies were different. The samples in Xu and Chang’s study were grown in the U.S. while all of the samples used in this study were grown in China.

The TFC of the red kidney bean and the black bean were also measured in a previous study [18] by employing the same method as mentioned in this study. However, the samples explored in the present were produced in China while the two legumes studied by Xu et al. [18] were cultivated in the U.S. The TFC values of the red kidney bean from Kunming, Yunnan and small black bean from Liangshan, Sichuan in this study were comparatively higher than the findings of Xu et al. [18] which may imply that the red kidney bean and black bean cultivated in China exhibit better antioxidant ability. Nevertheless, the flavonoid contents of legumes decrease as the storage time increases [19]. Thus, the discrepancy may also be because the legumes studied by Xu et al. were stored for a longer period of time before measurement. Besides, the small black bean also presents a significant variation in size compared to the size of a black turtle bean.

Condensed tannins, also known as proanthocyanidin, are the oligomers and polymers of flavonoids [20]. They are mainly located in the seed coat or testa of legumes. They can prevent plants from being attacked by microorganisms through deactivating aggressive enzymes, and also serve as a defense system for seeds to protect themselves against oxidative damage [21]. In addition, Elias et al. [22] reported that tannin is present in both whole seeds as well as seed coats, while the tannin content of some legumes with a deep color of seed coats was much higher than the whole seed. The color of the seed coats of 23 legumes measured in this experiment varied from very light and white to a complex speckled color. Velvet beans from Hechi, Guangxi exhibit the highest content for both TPC and TFC but contain a relatively low content of condensed tannin (2.84 mg CAE/g) because of the light color of its seed coat. Meanwhile, stone beans from Dazhou, Sichuan (12.95 mg CAE/g) exhibit the highest amount of condensed tannin that is attributed to its dark purple seed coat with black pattern. The white flat beans from Shangrao, Shanxi with lower color values present the lowest value for CTC (0.98 mg CAE/g).

### 4.2. Antioxidant Capacities of Under-Utilized Legumes

The FRAP method [13] was used in this study to determine the antioxidant ability of 23 legume samples. The reaction time of the FRAP reagent and each of the legume extracts was accurately controlled for six min. As shown in Table 1, the velvet bean from Hechi, Guangxi exhibited significantly higher FRAP values (102.8 mmol Fe^2+^ /100 g) compared to the other legume samples. Its antioxidant ability was 4.4-folds higher than the legume sample with the second highest FRAP value. However, the white flat bean from Shangrao, Shanxi (0.59 mmol Fe^2+^/100g) presented the lowest antioxidant ability as accessed by FRAP assay. The other legume samples exhibited significant FRAP values varying from 10.38 to 23.24 mmol Fe^2+^/100g, except for the large white kidney from Diqing Tibetan Autonomous Prefecture, Yunnan (0.90 mmol of Fe^2+^/100g), white kidney from Diqing Tibetan Autonomous Prefecture, Yunnan (0.78 mmol of Fe^2+^/100g) and small white bean from Chengdu, Sichuan (0.96 mmol of Fe^2+^/100g) that presented very low values for FRAP.

The FRAP values of the red kidney bean and small black bean were also reported earlier by Xu et al. [18]. Compared to the previous study, both the red kidney bean from Kunming, Yunnan (12.92 mmol of Fe^2+^/100g) and small black bean from Liangshan, Sichuan (15.33 mmol of Fe^2+^/100g) explored in this study revealed higher FRAP values, around 5 mmol of Fe^2+^/100g. The possible reasons for this difference could be the same as mentioned previously in that the variations in the FRAP values may also be attributed to the difference in the geographical origin of samples, the type of black bean and the storage time.

The FRAP method was not sufficient for studying the antioxidant ability of the legume extracts. Thus, the ABTS method was also used to measure the antioxidant ability of the legume samples. Compared to the FRAP method, the ABTS method is more sensitive. The ABTS radical generated by potassium persulfate acts as a great tool for measuring the antioxidant activity of hydrogen-donating antioxidants and chain-breaking antioxidants [23]. In an aqueous phase, the ABTS method determines the relative antioxidant power of legumes by scavenging ABTS radicals. The discoloration of ABTS indicates the potential capability of an oxidant [24]. Legumes with higher antioxidant ability present strong ABTS scavenging rates, thus leading to a lighter color of the aqueous solution.

Among the 23 types of legumes tested, the ABTS value of the velvet bean from Hechi, Guangxi (298.71 µmol TE/g) was found to be the highest one, while the white flat beans from Shangrao, Shanxi (5.41 µmol TE/g) possessed the lowest ABTS value. Large white kidney beans from Diqing Tibetan Autonomous Prefecture, Yunnan (9.04 µmol TE/g), white kidney beans from Diqing Tibetan Autonomous Prefecture, Yunnan (7.74 µmol TE/g) and small white beans from Chengdu, Sichuan (8.76 µmol TE/g) also presented very low values for ABTS. Except for these three legume samples, the other legume samples exhibited significant ABTS values, varying from 17.36 µmol TE/g to 298.71 µmol TE/g. These findings suggest that the extracts of legume samples are capable of preventing the oxidative damages from organic radicals.

In fact, the ABTS method also has its limitations. The selectivity of ABTS was not only poor towards the reaction with the hydrogen atom donors, but also reduced by the aromatic hydroxyl group that result in no contribution in anti-oxidation [25]. Hence, to gain a more comprehensive understanding of the antioxidant capacity of legumes, application of other methods such as DPPH or ORAC are suggested to be used together in future studies. Due to the different principles and reagents used in each method, the antioxidant capacity measured using these methods is not comparable. The antioxidant ability of each antioxidant can be expressed either as an amount of phenolic contents or the ability to scavenge radicals. The magnitude of the values of the legume samples measured with each method only represents the extent of their antioxidant ability as shown under the method.

### 4.3. Color Values

In this study, the seed coats and the cotyledon were ground together to obtain the whole bean powder. The color values were detected based on the whole bean powder. Large white kidney from Diqing Tibetan Autonomous Prefecture, Yunnan with white seed coat color showed the highest *L* value (99.17), while the small black bean from Liangshan, Sichuan showed the lowest *L* value (79.67). The small black bean also revealed the highest value for greenness (*b* = −4.13) and yellowness (*a =* 15.75). Meanwhile, the pinto bean from Kunming, Yunnan exhibited the highest value for redness (*a* = 3.74), whereas the stone bean from Dazhou, Sichuan presented the highest value for blueness (*b* = 0.9). There were significant differences in *L, a, b* values among most of the legume samples; no significant difference was observed in *L* values of the small zebra bean (Heze, Shandong) and broad bean (Kunming, Yunnan), in *a* values of the pinto bean (Kunming, Yunnan) and small zebra bean (Heze, Shandong) and in *b* values of the red pinto bean (Anqing, Anhui) and pinto kidney bean (Nujiang Lisu Autonomous Prefecture, Yunnan).

The findings of this study indicated that both TPC and TFC are well correlated with the antioxidant ability of 23 legumes. However, CTC didn’t contribute too much towards the antioxidant ability of the legumes. The results of the present study are slightly different from the previous findings of Xu et al. [18] that mentioned a good correlation among the three antioxidant assays, TPC, TFC and CTC. These variations in the findings of both studies may be attributed to the differences in the seed coats color of legume samples explored in both studies. Seed coats are the richest source of phenolic acids. It is reported that the phenolic acids of bean coats could vary from 146 to 5798 mg GAE/100 g [26]. However, the whole bean powder may decrease the overall value of phenolic acid in the sample to some extent.

### 4.4. Correlations between Phytochemicals, Antioxidant Capacities and Color Values of 23 Legumes

In this study, CTC exhibited a highly negative correlation with *L* value (*r* = −0.637). This revealed that the legumes with light color contain less quantity of CTC. Elias et al. [22] discovered that the legumes with deep color have more condensed tannin in their seed coat than in the whole seed. Another study by Caldas and Blair [27] also reported that darker color legumes usually contain higher CTC. There were no obvious correlations among the color values (*L*, *a*, *b*) of all legumes.

A moderate negative correlation between TPC and *L* value (*r* = −0.433), and ABTS and *L* value (*r* = −0.518) was found at the level of 0.05, but no obvious correlation was observed between FRAP and *L* value. This indicated that the phenolic content and antioxidant ability of legumes might have a moderate correlation with their color. These findings are similar to a previous study that mentioned a high level of TPC and CTC in case of beans with dark color seed coats and a low amount of TPC and CTC in the case of beans with white and yellow seed coats [28]. Another study has also reported a high level of TPC in the case of violet rice flour compared to other types of rice flours [29] that, in turn, proved the correlation between the phenolic acids and the color of the grains.

In accordance with a previous study [30], the beans with light color contained a significantly less amount of phenolics than the colored beans. However, this trend might not be consistent. It was reported that the variations in phenolic acids may also be attributed to the types of legumes. For example, the adzuki bean from Mudanjiang, Heilongjiang with an *L* value of 86.31 and the pinto bean from Kunming, Yunnan with an *L* value of 91.93 exhibited a comparable amount of TPC around 8.9 mg GAE/g. Similar results were also reported by Xu and Chang [31].

### 4.5. Free Phenolic Acids in Legume Samples

As shown in Table 4, gallic acid was identified in most of the samples except the small zebra bean from Heze, Shandong. Gallic acid was also found be a major phenolic acid among all legume samples as its quantity was relatively higher compared to other free phenolic acids. However, according to Xu & Chang [31], a higher amount of chlorogenic acid was detected in food legumes compared to gallic acid, whereas the higher amount of sinapic acid was found in lentil. Chlorogenic acid was also found in a higher amount compared to gallic acid [32]. Gallic acid was also found as the only phenolic acid in broad bean [33] while nine types of phenolic acids were determined in broad beans from Kunming, Yunnan in this experiment. Various factors such as the different varieties, cultivation area and storage time may contribute towards the variations in the free phenolic acids mentioned in different studies [34]. However, the gallic acid content of the broad bean (24.55 μg/g) was found to be similar to one of the broad beans (22.9 μg/g) reported by Magalhães et al. [33]. A small quantity of ferulic acid was detected in 14 legume samples. This finding is in agreement with a previous study reported by Xu and Chang [31,32].

In this study, *p*-hydroxybenzoic acid, vanillic acid, *p*-coumaric acid, gentistic acid, ferulic acid, 2,3,4-trihydroxybenzoic acid, syringic acid, protocatechualdehyde and vanillin were detected in more than half of the legume samples. The sinapic acid was only detected in 10 samples. However, according to the report of Luthria & Pastor-Corrales [35], *p*-coumaric acid, ferulic acid and sinapic acid were identified in all of the 15 legumes samples explored in that study. However, the legume samples explored by Luthria & Pastor-Corrales [35] were from America. The significant variations in the results of various studies clearly represent the impact of geographical area on the phytochemical profile of particular plant material. In this study, salicylic acid was observed to be the least predominant free phenolic acid that was only found in four out of 23 legume samples.

The free phenolic acids profile varies significantly among all of the legume samples. Adzuki beans from Mudanjiang, Heilongjiang and milky flower beans from Luohe, Henan were observed to contain 12 types of free phenolic acid. However, adzuki beans contained relatively higher amounts of vanillin (72.97 μg/g) compared to other free phenolic acids detected in this legume. While in the case of milky flower beans, a comparable amount of 12 free phenolic acids was detected. Large zebra beans from Shaoguan, Guangdong and velvet beans from Hechi, Guangxi also contain various phenolic acids. In the case of the large zebra bean, a relatively high content of vanillic acid was detected and only a small quantity of protocatechualdehyde was detected. Among 23 legume samples, velvet beans exhibited the highest level of gallic acid (479.26 μg/g), vanillin (219.19 μg/g) and protocatechualdehyde (29.71 μg/g). In this study, velvet beans also revealed the highest total phenolic content and highest antioxidant activity accessed by ferric reducing antioxidant capacity (FRAC) assay and ABTS free radical scavenging activity (ABTS) assay. In view of this, the high level of TPC and antioxidant ability of the velvet bean may be attributed to its high concentration of gallic acid, vanillin and protocatechualdehyde. The small white bean from Chengdu, Sichuan presented low levels of TPC and low antioxidant capacity which was in accordance with the presence of only gallic acid (14.52 μg/g). Other legume samples under investigation contained 4-10 types of phenolic acid.

### 4.6. Flavonoids in Legume Samples

Flavonoid contents of all 23 legume samples were measured by HPLC and summarized in Table 5. Seven types of flavonoid were detected in 23 samples explored in this study. Among these seven types of flavonoids, hesperitin, tangeritin and quercitrin and hesperidin were the most common flavonoids in legume samples. However, in Hempel and Böhm’s study [36], kaempferol and quercetin were found to be the main flavonoid, while in this study kaempferol was not detected in any of the legume samples, but a small quantity of quercetin was detected only in small black beans from Liangshan, Sichuan. In Romani’s study [37], kaempferol was reported to be present in the legumes. Espinosa-Alonso et al. [34] have also reported that quercetin and kaempferol are the main flavonoids in their legume samples. This difference in the flavonoid profiles of legumes may be attributed to the variation in cultivation area and varieties of samples investigated in various studies.

The major flavonoids such as flavonols, flavones and anthocyanins are widely present in edible plants, in which flavonols usually present as glycosides such as myricetin, quercetin, kaempferol, isorhamnetin and rhamnetin [38]. Bean hulls contain significant amounts of phytochemicals that contribute towards the high antioxidant activity of these beans [39]. The high concentration of anthocyanins is associated with the black or purple seed coat color of *P. vulgaris* [16]. In this study, the small black bean was found to have the highest value for the sum of flavonoids and also found to contain various types of flavonoids. Hesperitin, quercitrin and hesperidin, myricetin, morin, quercetin and luteolin, galangin and tangeretin were detected in small black beans from Liangshan, Sichuan, among which the content of hesperitin, myricetin and morin were much higher compared to the other legumes. It is also interesting to note that quercetin and luteolin were only found in the small black bean. The high content and presence of different types of flavonoids may be attributed to the deep black color of its seed coat.

Small adzuki beans from Jixi, Heilongjiang (149.86 μg/g) revealed the second highest value for the sum of flavonoids and also ranked second to exhibit high variation in types of flavonoids detected. Hesperitin, naringin, quercitrin and hesperidin, morin and tangeritin were also detected in small adzuki beans. Small adzuki beans present the highest amount of naringin, quercitrin and hesperidin compared to other legume samples explored in this study, whereas in the case of the adzuki bean, hesperitin (7.71 μg/g) was the only flavonoid detected. It was also found that the small adzuki bean also exhibited a higher level of total phenolics, higher total flavonoids and higher antioxidant ability than the adzuki bean from Mudanjiang, Heilongjiang.

## 5. Conclusions

In conclusion, 21 under-utilized food legumes investigated in this study presented significant variation in their phenolic profile and their antioxidant ability. Velvet beans from Hechi, Guangxi exhibited the highest value of total phenolic, total flavonoid, and free phenolic acids and showed the strongest antioxidant ability among the 21 under-utilized food legumes and two reference bean samples. The white flat bean from Shangrao, Shanxi presented the lowest value for both the phytochemicals and antioxidants. Gallic acid was observed to be the most predominant free phenolic acid and hesperitin was the main flavonoid in legume samples under investigation. Significant differences were observed in the phenolics and antioxidant activities of most legumes. Correlation analysis found that the antioxidant ability was moderately related to the color of legumes, and highly related to the total phenol and flavonoid content of legumes. Moreover, in this study only two adzuki beans from Mudanjiang, Heilongjiang and Jixi, Heilongjiang have been used as a reference material. Furthermore, to achieve a comprehensive assessment of phytochemical profile and antioxidant activity of samples under investigation, multiple selections have to be done in order to standardize the starting material. In addition, along with the free phenolic acids and flavonoids, bound phenolic acids, bioactive peptides of legume samples and their bioavailability will be explored in future.

## Figures and Tables

**Table 1:** Total phenolic content (TPC), total flavonoid content (TFC), condensed tannin content (CTC) and antioxidant capacities (ABTS and Ferric Reducing Antioxidant Capacity (FRAP) values) of 23 food legumes.

ID	English Name	Scientific Name	TPC	TFC	CTC	FRAP	ABTS
			(mg GAE/g)	(mg CAE/g)	(mg CAE/g)	(mmol of Fe^2+^E/100 g)	(μmol TE/g)
1	Small adzuki bean	*Vigna umbellata*	13.29 ± 0.21 c	7.91 ± 0.04 c	9.47 ± 0.05 c	19.27 ± 0.68 c	104.20 ± 3.08 d
2	Adzuki bean	*Vigna angularis* (Willd.)	8.91 ± 0.16 fg	5.85 ± 0.09 fgh	7.03 ± 0.35 g	11.81 ± 0.61 fg	72.39 ± 1.12 j
3	Pinto bean	*Phaseolus vulgaris*	8.97 ± 0.21 fg	5.58 ± 0.01 h	7.72 ± 0.33 f	11.59 ± 0.67 gh	70.96 ± 1.42 j
4	Red pinto bean	*Phaseolus vulgaris*	8.22 ± 0.38 h	5.28 ± 0.21 i	6.92 ± 0.31 g	10.38 ± 0.47 hi	66.08 ± 1.43 k
5	Milky flower bean	*Phaseolus vulgaris*	5.18 ± 0.17 k	2.82 ± 0.06 m	4.93 ± 0.26 i	6.64 ± 0.16 j	39.59 ± 1.64 n
6	Stone bean	*Flemingia fluminalis* C.B. Clarke	13.27 ± 0.68 c	6.40 ± 0.07 e	12.95 ± 0.20 a	23.23 ± 0.67 b	122.66 ± 0.70 c
7	Light speckled bean	*Phaseolus vulgaris*	2.37 ± 0.09 l	2.66 ± 0.08 m	3.02 ± 0.18 j	2.99 ± 0.09 k	17.36 ± 1.01 o
8	Spotted cowpea	*Vigna unguiculata*	14.62 ± 0.45 b	10.39 ± 0.39 b	12.02 ± 0.55 b	23.24 ± 0.57 b	124.87 ± 2.08 c
9	Large zebra bean	*Phaseolus coccineus* Linn.	6.79 ± 0.13 i	4.74 ± 0.13 j	5.72 ± 0.22 h	9.53 ± 0.40 i	58.69 ± 4.17 l
10	White flat bean	*Dolichos lablab* L.	0.71 ± 0.06 m	1.05 ± 0.01 p	0.98 ± 0.02 m	0.59 ± 0.01 l	5.41 ± 0.12 p
11	Pinto kidney bean	*Phaseolus vulgaris*	8.57 ± 0.35 gh	5.65 ± 0.14 gh	7.73 ± 0.28 f	11.45 ± 0.43 gh	77.04 ± 2.22 i
12	Red kidney	*Phaseolus vulgaris*	9.18 ± 0.18 ef	5.80 ± 0.08 fgh	8.44 ± 0.28 e	12.92 ± 0.11 f	79.38 ± 1.10 hi
13	Large white kidney bean	*Phaseolus vulgaris* Linn.	1.04 ± 0.02 m	1.34 ± 0.06 o	1.61 ± 0.07 l	0.90 ± 0.01 l	9.04 ± 0.15 p
14	Small round pinto bean	*Phaseolus vulgaris*	6.17 ± 0.06 j	4.39 ± 0.10 k	5.81 ± 0.08 h	9.66 ± 0.74 i	53.95 ± 1.43 m
15	Small zebra bean	*Phaseolus coccineus* Linn.	9.62 ± 0.40 e	7.11 ± 0.26 d	8.57 ± 0.41 de	15.48 ± 0.36 e	86.38 ± 3.60 f
16	Velvet bean	*Mucuna cochinchinesis* (Lour) Tang	56.63 ± 0.88 a	34.50 ± 0.48 a	2.84 ± 0.02 j	102.82 ± 3.07 a	298.71 ± 3.09 a
17	Broad bean	*Vicia faba* L.	9.56 ± 0.29 e	4.09 ± 0.16 l	8.82 ± 0.34 de	18.83 ± 0.14 c	140.55 ± 2.38 b
18	White kidney bean	*Phaseolus vulgaris*	0.74 ± 0.03 m	1.46 ± 0.04 o	2.28 ± 0.06 k	0.78 ± 0.03 l	7.74 ± 0.39 p
19	Green adzuki bean	*Vigna umbellata*	10.84 ± 0.16 d	5.91 ± 0.14 fg	8.96 ± 0.26 d	17.38 ± 0.58 d	96.88 ± 1.49 e
20	Black-eyed pea	*Vigna unguiculata*	2.34 ± 0.11 l	2.25 ± 0.05n	2.68 ± 0.13 jk	3.88 ± 0.05 k	19.64 ± 1.31 o
21	Small white bean	*Phaseolus vulgaris* L. f.abla Alef	0.74 ± 0.02 m	1.27 ± 0.03op	1.52 ± 0.07 l	0.96 ± 0.04 l	8.76 ± 0.29 p
22	Mosaic bean	*Vigna unguiculata* (Linn.) Walp	11.16 ± 0.60 d	7.29 ± 0.02d	8.41 ± 0.25 e	16.94 ± 0.37 d	81.49 ± 4.85 gh
23	Small black bean	*Phaseolus vulgaris*	12.79 ± 0.04 c	6.03 ± 0.09f	7.66 ± 0.11 f	15.33 ± 0.46 e	84.53 ± 3.53 fg

Data were expressed as mean ± standard deviation (*n* = 3). The data in the same column marked with different small case letters are significantly (*p* < 0.05) different.

**Table 2:** Color value (*L*, *a*, *b*) of 23 food legumes.

ID Code	English Name	*L*	*a*	*b*
1	Small adzuki bean	81.28 u	1.67 k	3.66 n
2	Adzuki bean	86.31 r	2.32 g	1.32 t
3	Pinto bean	91.93 h	3.74 a	3.07 p
4	Red pinto bean	88.71 p	3.28 c	1.35 s
5	Milky flower bean	91.83 i	1.92 i	1.79 r
6	Stone bean	83.90 t	3.49 b	0.90 v
7	Light speckled bean	96.13 e	0.73 n	5.92 f
8	Spotted cowpea	89.11 n	1.70 j	4.45 j
9	Large zebra bean	86.08 s	2.92 e	3.99 l
10	White flat bean	98.94 b	−1.29 u	8.46 d
11	Pinto kidney bean	91.49 l	2.96 d	1.34 s
12	Red kidney	93.32 f	1.96 h	1.23 u
13	Large white kidney bean	99.17 a	−1.02 t	8.55 c
14	Small round pinto bean	91.59 k	2.73 f	3.83 m
15	Small zebra bean	89.42 m	3.73 a	2.96 q
16	Velvet bean	87.52 q	1.06 m	7.73 e
17	Broad bean	91.59 k	−0.35 s	9.93 b
18	White kidney bean	96.56 d	0.39 o	5.28 h
19	Green adzuki bean	92.52 g	0.18 r	4.01 k
20	Black-eyed pea	91.65 j	0.27 p	5.70 g
21	Small white bean	97.11 c	0.20 q	3.32 o
22	Mosaic bean	89.01 o	1.17 l	4.6 i
23	Small black bean	79.67 v	−4.13 v	15.75 a

Data were expressed as mean ± standard deviation (*n* = 3). The data in the same column marked with different letters are significantly (*p* < 0.05) different.

**Table 3:** Correlation between phenolics (TPC, TFC, CTC), antioxidant capacity (FRAP, ABTS), and color values by 23 legumes.

Variables	Correlation	TPC	TFC	CTC	FRAP	ABTS	*L*	*a*	*b*
TPC	Pearson Correlation	1	0.990 b	0.161	0.995 b	0.944 b	-0.433a	0.074	0.131
	Sig.( 2-tailed)		0	0.463	0	0	0.039	0.737	0.55
	*N*		23	23	23	23	23	23	23
TFC	Pearson Correlation		1	0.104	0.987 b	0.912 b	−0.363	0.105	0.104
	Sig.(2-tailed)			0.636	0	0	0.088	0.633	0.638
	*N*			23	23	23	23	23	23
CTC	Pearson Correlation			1	0.118	0.412	−0.637 b	0.395	−0.286
	Sig.(2-tailed)				0.593	0.051	0.001	0.062	0.187
	*N*				23	23	23	23	23
FRAP	Pearson Correlation				1	0.940 b	−0.371	0.069	0.134
	Sig.(2-tailed)					0	0.081	0.753	0.543
	*N*					23	23	23	23
ABTS	Pearson Correlation					1	−0.518 a	0.144	0.089
	Sig.(2-tailed)						0.011	0.512	0.685
	*N*						23	23	23
*L*	Pearson Correlation						1	−0.141	−0.043
	Sig.(2-tailed)							0.522	0.846
	*N*							23	23
*a*	Pearson Correlation							1	−0.870 b
	Sig.(2-tailed)								0
	*N*								23
*b*	Pearson Correlation								1
	Sig.(2-tailed)								
	*N*								

a Correlation is significant at the 0.05 level (2-tailed), b Correlation is significant at the 0.01 level (2-tailed).

**Table 4:** The free phenolic acid contents (µg/g) of legume samples.

Sample No.	English Name	GA	PA	TBA	PCD	HBA	GEA	CLA	VA	SA	VN	PCA	FA	SIA	SAA	Total
1	Small Adzuki bean	10.53 ± 0.22def	ND	2.64 ± 0.05c	1.81 ± 0.11cd	6.67 ± 0.03a	ND	ND	ND	1.68 ± 0.14gh	87.85 ± 7.06c	1.89 ± 0.03cdef	ND	ND	ND	113.07
2	Adzuki bean	9.74 ± 0.47ef	ND	1.74 ± 0.15e	1.86 ± 0.04cd	0.59 ± 0.22ij	5.22 ± 0.03e	1.98 ± 0.09ef	3.76 ± 0.1g	0.57 ± 0.02i	72.97 ± 2.04d	2.26 ± 0.04cde	1.48 ± 0.04c	ND	0.59 ± 0.01d	102.76
3	Pinto bean	8.47 ± 0.14ef	0.25 ± 0d	1.96 ± 0.02d	7.84 ± 0.18b	4.07 ± 0.16cd	ND	ND	2.07 ± 0.05h	1.38 ± 0.07h	ND	ND	ND	0.64 ± 0.02def	ND	26.68
4	Red pinto bean	7 ± 0.05f	0.16 ± 0d	1.91 ± 0.02d	ND	4.24 ± 0.09c	ND	ND	3.53 ± 0.18g	5.53 ± 0.37b	ND	2.2 ± 0.04cde	4.23 ± 0.15c	0.34 ± 0.01ef	ND	29.14
5	Milky flower bean	9.24 ± 0.41ef	1.85 ± 0.04bc	1.72 ± 0.01e	6.27 ± 0.1b	2.11 ± 0.16f	ND	0.5 ± 0.02ghi	3.79 ± 0.2g	2.42 ± 0.05ef	3.06 ± 0.14hi	2.13 ± 0.09cde	2.16 ± 0.11c	0.5 ± 0.03ef	ND	35.75
6	Stone bean	11.95 ± 0.42de	0.52 ± 0.01d	1.74 ± 0e	1.03 ± 0.08cd	2.29 ± 0.21f	0.81 ± 0.04i	0.5 ± 0.02d	ND	ND	ND	ND	2.7 ± 0.09c	ND	ND	21.54
7	Light speckled bean	12.39 ± 0.15de	ND	2.01 ± 0.02d	ND	1.36 ± 0.1g	5.82 ± 0.17cd	ND	4.69 ± 0.1f	ND	5.46 ± 0.16ghi	1.62 ± 0.05cdefg	3.19 ± 0.04c	0.21 ± 0.01f	ND	36.75
8	Spotted cowpea	9.48 ± 0.16ef	ND	2.76 ± 0.21c	8.63 ± 0.39b	ND	18.94 ± 0.34a	ND	ND	ND	4.35 ± 0.36ghi	1.44 ± 0.09defgh	ND	ND	ND	45.6
9	Large zebra bean	7.25 ± 0.06f	4.68 ± 0.07a	1.58 ± 0.06f	0.11 ± 0d	3.89 ± 0.17d	5.59 ± 0.22de	ND	12.36 ± 0.11c	3.48 ± 0.04d	1.16 ± 0.02i	1.71 ± 0.05cdefg	ND	0.81 ± 0.06def	ND	42.62
	White flat beans	8.98 ± 0.16ef	1.09 ± 0.04bcd	ND	1.02 ± 0.02cd	0.21 ± 0.0kl	ND	20.69 ± 0.04a	14.85 ± 0.05a	ND	23.94 ± 2.3f	5.88 ± 0.29b	2.2 ± 0.03c	0.27 ± 0ef	ND	79.13
11	Pinto kidney bean	9.13 ± 0.14ef	ND	1.67 ± 0.02ef	ND	2.78 ± 0.07e	ND	1.12 ± 0.11fgh	5.44 ± 0.08e	ND	ND	1.3 ± 0.04efgh	2 ± 0.01c	0.27 ± 0.01ef	ND	23.71
12	Red kidney	12.26 ± 0.18de	2.23 ± 0.16b	1.7 ± 0.07ef	ND	4.13 ± 0.16cd	0.64 ± 0.02ij	ND	5.48 ± 0.17e	ND	ND	0.33 ± 0.02gh	17.98 ± 0.21b	0.38 ± 0.02ef	ND	45.13
13	Large kidney bean	0.39 ± 0g	ND	ND	ND	ND	3.15 ± 0.17f	ND	8.85 ± 0.06d	6.08 ± 0.05a	8.86 ± 0.09ghi	ND	ND	1.36 ± 0.03cde	ND	28.69
14	Small round pinto bean	1.99 ± 0.15g	1.28 ± 0.03bcd	ND	ND	ND	7.11 ± 0.41b	ND	2.08 ± 0.08h	3.67 ± 0.11d	43.74 ± 0.21e	ND	0.59 ± 0.04c	1.94 ± 0.01c	1.56 ± 0.11bc	63.96
15	Small zebra bean	ND	1.04 ± 0.04bcd	ND	ND	6.9 ± 0.48a	2.21 ± 0.06g	ND	2.18 ± 0.06h	3.42 ± 0.09d	23.97 ± 0.82f	ND	1.76 ± 0.05c	ND	12.55 ± 0.68a	54.03
16	Velvet bean	479.26 ± 10.4a	ND	6.13 ± 0.16a	29.71 ± 0.96a	5.69 ± 0.12b	6.31 ± 0.01c	2.45 ± 0.04e	2.09 ± 0.02h	0.32 ± 0.01i	219.19 ± 7.8a	3.01 ± 0.09c	ND	ND	1.8 ± 0.04b	755.96
17	Broad bean	24.55 ± 0.23c	0.31 ± 0.02d	ND	1.07 ± 0.05cd	1.08 ± 0.05h	0.11 ± 0.01jk	ND	0.33 ± 0.02j	2.46 ± 0.06ef	161.63 ± 3.74b	ND	3.13 ± 0.04c	ND	ND	194.67
18	White kidney bean	10.1 ± 0.43ef	ND	ND	1.07 ± 0.05cd	ND	2.2 ± 0.14g	ND	ND	ND	ND	2.94 ± 0.2cd	1.37 ± 0.01c	ND	ND	17.68
19	Green adzuki bean	12.25 ± 0.21de	0.78 ± 0.02cd	5.81 ± 0.01b	3.09 ± 0.11c	ND	2.12 ± 0.02h	ND	ND	ND	ND	1.55 ± 0.11cdefg	3.1 ± 0.1c	ND	ND	28.7
20	black-eyed pea	12.17 ± 0.21de	ND	ND	1.45 ± 0.07cd	0.36 ± 0.01jk	2.2 ± 0.05g	ND	0.14 ± 0.01j	0.2 ± 0.01i	ND	ND	2.94 ± 0.24c	ND	ND	19.46
21	Small white bean	14.52 ± 0.43d	ND	ND	ND	ND	ND	ND	ND	ND	ND	ND	ND	ND	ND	14.52
22	Mosaic bean	11.07 ± 0.28def	0.8 ± 0.04cd	ND	ND	ND	ND	13.3 ± 0.09b	ND	ND	ND	1.62 ± 0.08cdefg	ND	ND	ND	26.79
23	Small black bean	28.64 ± 0.03b	ND	ND	ND	ND	ND	ND	ND	2 ± 0.08fg	ND	6.53 ± 0.42b	ND	ND	ND	37.17

Data are expressed as mean ± standard deviation (*n* = 3). The data in the same column marked with different small case letters are significantly (*p* < 0.05) different. Phenolic acid: GA: gallic acid; PA: protocatechuic acid; TBA: 2,3,4-trihydroxybenzoic acid; PCD: protocatechualdehyde; HBA: *p*-hydroxybenzoic acid; GEA: gentistic acid; CLA: chlorogenic acid; VA: vanillic acid; SA: syringic acid; VN: vanillin; PCA: *p*-coumaric acid; FA: ferulic acid; SIA: sinapic acid; SAA: salicylic acid; ND: not detectable.

**Table 5:** The flavonoids content (µg/g) of legume samples.

Sample No.	English Name	Hesperitin	Naringin	Q1	Myricetin	Morin	Q2	Tangeritin	Total
1	Small adzuki bean	34.23 ± 1.72 b	76.79 ± 5.78 a	21.69 ± 0.06 a	ND	11.93 ± 0.38 b	ND	5.22 ± 0.52 cd	149.86
2	Adzuki bean	7.71 ± 0.58 ij	ND	ND	ND	ND	ND	ND	7.71
3	Pinto bean	ND	ND	ND	ND	ND	ND	3.62 ± 0.02 e	3.62
4	Red pinto bean	18.38 ± 0.38 f	13.88 ± 1.11 f	ND	ND	ND	ND	4.92 ± 0.28 d	37.18
5	Milky flower bean	23.18 ± 0.16 e	44.99 ± 1.35 c	3.83 ± 0.26 h	ND	ND	ND	ND	72
6	Stone bean	5.99 ± 0.43 jk	ND	ND	ND	ND	ND	ND	5.99
7	Light speckled bean	4.56 ± 0.54 k	ND	ND	ND	ND	ND	ND	4.56
8	Spotted cowpea	9.02 ± 0.63 i	ND	11.51 ± 0.01 d	ND	ND	ND	ND	20.53
9	Large zebra bean	14.87 ± 1.94 g	ND	ND	ND	ND	ND	ND	14.87
10	White flat beans	9.77 ± 0.11 i	ND	ND	ND	ND	ND	4.12 ± 0.06 e	13.89
11	Pinto kidney bean	13.71 ± 0.03 gh	ND	ND	ND	ND	ND	5.26 ± 0.19 bcd	18.97
12	Red kidney	27.92 ± 0.16 d	ND	4.34 ± 0.02 g	ND	ND	ND	5.63 ± 0.04 bc	37.89
13	Large kidney bean	ND	ND	ND	ND	ND	ND	2.71 ± 0.05 f	2.71
14	Small round pinto bean	ND	ND	ND	ND	ND	ND	2.69 ± 0.08 f	2.69
15	Small zebra bean	24.22 ± 0.53 e	ND	4.25 ± 0.4 gh	ND	ND	ND	ND	28.47
16	Velvet bean	13.28 ± 0.94 gh	ND	ND	ND	ND	ND	ND	13.28
17	Broad bean	12.26 ± 0.65 h	16.97 ± 0.36 e	4.84 ± 0.07 f	ND	ND	ND	ND	34.07
18	White kidney bean	4.81 ± 0.02 k	ND	ND	ND	ND	ND	ND	4.81
19	Green adzuki bean	28.59 ± 2.19 d	22.02 ± 1.05 d	7.73 ± 0.49 e	ND	ND	ND	ND	58.34
20	black-eyed pea	32.1 ± 2.59 c	57.42 ± 3.13 b	ND	ND	ND	ND	ND	89.52
21	Small white bean	ND	ND	ND	ND	ND	ND	5.8 ± 0.51 b	5.8
22	Mosaic bean	26.97 ± 1.31 d	ND	13.41 ± 0.01 c	6.12 ± 0.2 b	ND	ND	ND	46.5
23	Small black bean	38.1 ± 1.15 a	ND	16.7 ± 0.81 b	63.32 ± 2.2 a	39.27 ± 2.26 a	5.8 ± 0.4 a	6.65 ± 0.91 a	169.84

Data are expressed as mean ± standard deviation (*n* = 3). The data in the same column marked with different small case letters are significantly (*p* < 0.05) different. Flavonoids: Q1: quercitrin + Hesperidin; Q2: quercetin + luteolin; ND: not detectable.

## Supplementary Materials

The following are available online at https://www.mdpi.com/2304-8158/9/4/438/s1, Table S1: Sample ID, Chinese names, English names, scientific names, moisture content, morphology, and sources of 23 legumes.
