# Supplementary material for: An Investigation on Phenolic and Antioxidant Capacity of Under-utilized Food Legumes Consumed in China
Source: Foods. 2020 Apr 6;9(4):438. doi: 10.3390/foods9040438 (PMC7231270; doi:10.3390/foods9040438)
Supplement: Supplementary file 1 [file foods-09-00438-s001.pdf]

Supplementary

Table S1. Sample ID, Chinese names, English names, scientific names, moisture content, morphology, and sources of 23 legumes.

Sample ID	Chinese name	English name	Scientific name	Moisture content	Morphology	Source
1	Chixiaodou	Small adzuki bean	*Vigna umbellata*	12.4 %	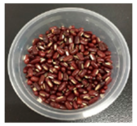	Commercial product
2	Chidou	Adzuki bean	*Vigna angularis* (Willd.) Ohwi et Ohashi	11.1 %	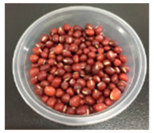	Commercial product
3	Quedandou	Pinto bean	*Phaseolus vulgaris*	13.2 %	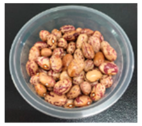	Commercial product
4	Jinsidou	Red pinto bean	*Phaseolus vulgaris*	10.9 %	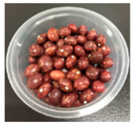	Commercial product
5	Naihuadou	Milky flower bean	*Phaseolus vulgaris*	13.3 %	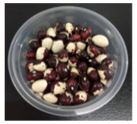	Commercial product
6	Yandou	Stone bean	*Flemingia fluminalis* C.B.Clarke	11.1 %	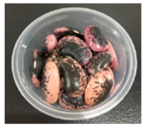	Commercial product
7	Bai huayaodou	Light speckled bean	*Phaseolus vulgaris*	13.1 %	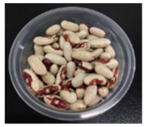	Commercial product

8	Majiangdou	Spotted cowpea	*Vigna unguiculata*	11.2 %	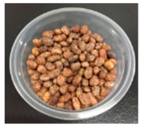	Commercial product
9	Hebaodou	Large zebra bean	*Phaseolus coccineus* Linn.	12.9 %	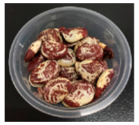	Commercial product
10	Baibiandou	White flat bean	*Dolicho lablabL.*	11.4 %	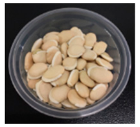	Commercial product
11	Huayaodou	Pinto kidney bean	*Phaseolus vulgaris*	12.6 %	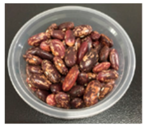	Commercial product
12	Hongyaodou	Red kidney	···*Phaseolus vulgaris*	11.4 %	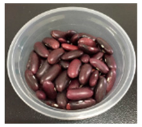	Commercial product
13	Baiyundou	Large white kidney bean	*Phaseolus vulgaris* Linn.	11.6 %	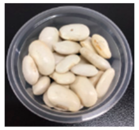	Commercial product
14	Yuan banmadou	Small round pinto bean	*Phaseolus vulgaris*	10.4 %	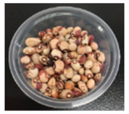	Commercial product
15	Banmadou	Small zebra bean	*Phaseolus coccineus* Linn.	9.9 %	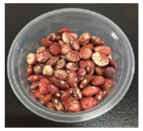	Commercial product

16	Maodou	Velvet bean	*Mucuna cochinchine-sis*(Lour)Tang et Wang	11.7 %	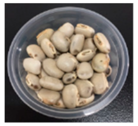	Commercial product
17	Chandou	Broad bean	*Vicia faba L.*	11.3 %	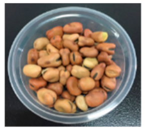	Commercial product
18	Baiyaodou	White kidney bean	*Phaseolus vulgaris*	13.3 %	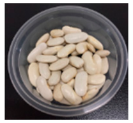	Commercial product
19	Zhudou	Green aduzki bean	*Vigna umbellata*	11.1 %	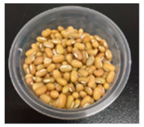	Commercial product
20	Mei dou	Black-eyed pea	*Vigna unguiculata*	11.8 %	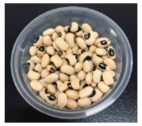	Commercial product
21	Xiaobaidou	Small white bean	*Phaseolus vulgaris L.* f.abla Alef	12.0%	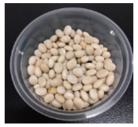	Commercial product
22	Huajiangdou	Mosaic bean	*Vigna unguiculata* (Linn.)Walp	11.3 %	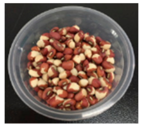	Commercial product
23	Xiaoheidou	Small black bean	*Phaseolus vulgaris*	10.0 %	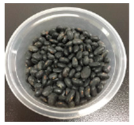	Commercial product
